# High-Speed Micro-Particle Motion Monitoring Based on Continuous Single-Frame Multi-Exposure Technology

**DOI:** 10.3390/ma15113871

**Published:** 2022-05-29

**Authors:** Wei Wang, Weiwei Xue, Shufan Wu, Zhongcheng Mu, Jiyuan Yi, Andrew J. Tang

**Affiliations:** 1 School of Aeronautics and Astronautics, Shanghai Jiao Tong University, Shanghai 200240, China; wangwei_up@sjtu.edu.cn (W.W.); shufan.wu@sjtu.edu.cn (S.W.); sjtuyjy@sjtu.edu.cn (J.Y.); 2 CAS Key Laboratory of Mechanical Behavior and Design of Material, Department of Modern Mechanics, University of Science and Technology of China, Hefei 230027, China; xueww@mail.ustc.edu.cn; 3 School of Aerospace, Mechanical and Mechatronic Engineering, The University of Sydney, Camperdown, NSW 2006, Australia; andrew.tang@sydney.edu.au

**Keywords:** micro-particles’ impact, motion monitoring, single-frame multiple-exposure, high-frequency light-emitting diode flash

## Abstract

The impact phenomena of solid micro-particles have gathered increasing interest across a wide range of fields, including space debris protection and cold-spray additive manufacturing of large, complicated structures. Effective motion monitoring is essential to understanding the impact behaviors of micro-particles. Consequently, a convenient and efficient micro-particle motion monitoring solution is proposed based on continuous single-frame multiple-exposure imaging technology. This method adopts a camera with excellent low-light performance coupled with high-frequency light-emitting diode (LED) flashes to generate short interval illumination. This technology can, in theory, achieve 1 million effective frames per second (fps) and monitor particles as small as 10 microns with speeds up to 12 km/s. The capabilities of the proposed method were validated by a series of micro-particle motion monitoring experiments with different particles sizes and materials under varying camera configurations. The study provides a feasible and economical solution for the velocity measurement and motion monitoring of high-speed micro-particles.

## 1. Introduction

**Figure 1:**
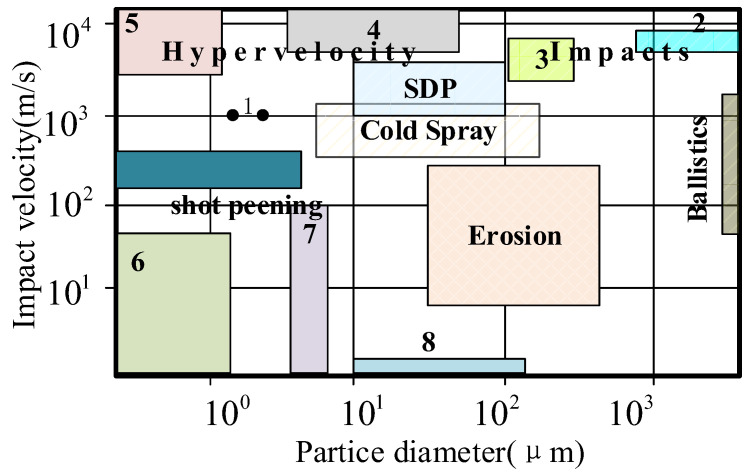
Impact phenomenon of solid particles.

The impact phenomena of solid particles are of interest to researchers across many fields, including the biomedicine, aerospace, and materials industries, among others. This phenomena can be divided into several key categories according to the velocity and size of the particle under observation: particles’ adhesion [1,2]; shot peening, including traditional pneumatic shot peening [3] (surface-strengthening phenomenon that occurs when particle diameters range between 0.1 and 1.0 μm and have an impact velocity less than 100 m/s) and supersonic shot peening (surface-strengthening phenomenon that occurs when particle diameters range between 0.1 and 1.0 μm and have an impact velocity greater than that of sound) [4,5,6,7]; particles’ surface erosion (dynamic process of surface material loss caused by the impact of particles with diameters of about 50–300 μm at speeds 10 to 300 m/s from differing impact angles) [8,9,10,11]; high-speed impact (impact phenomenon of particles with diameters ranging between 1 and 100 mm and velocity ranging between 50 and 3000 m/s) [12,13]; hypervelocity impact (impact velocity exceeding the speed of sound propagates in the two impact bodies, creating a temporary high-density and high-temperature state at the point of impact, leading to 1 TPa of pressure and vaporization of the associated materials) [14,15]; particles with super-deep penetration (SDP) (metal particles accelerated to 1–3 km/s impact the metal surface, resulting in a penetration depth between 1000 and 10,000 times the particles’ diameters) [16,17]; and cold spraying (particles’ deposition with a diameter of 5–150 μm at a velocity of 300–1200 m/s) [18,19,20], as shown in Figure 1.

Motion monitoring is necessary to investigate and understand the micro-particle impact behaviors which are observed across the many fields. In the research of space debris protection [14], the impacts of dust and space debris on large flexible parts like solar panels in space threaten the operation and safety of spacecraft. By monitoring the motion of micro-particles, an evaluation of initial impact conditions can be made, providing an important reference for spacecraft to minimize or avoid critical damage. For the biomedical industry, different velocities of drug micro-particles may lead to different subcutaneous injection depths with respect to needle-free injection [19]. On the one hand, different drug micro-particles have different requirements for the depth of skin invasion due to their different mechanisms and effects. On the other hand, there is an optimal range of subcutaneous depth for the same drug, which can be better absorbed by the body. Therefore, accurate movement monitoring is beneficial for improving drug utilization efficiency. In cold spray additive manufacturing [18,20], it is of great importance to accurately measure the velocity of micro-particles in order to understand the corresponding relationship between the state of micro-particles and the outcomes of cold spraying. Micro-particle sizes in the above fields may be as small as a few microns, while reaching speeds of up to a few kilometers per second or more. The extreme characteristics in these fields make monitoring the motion of micro-particles difficult to achieve.

In understanding the impact behaviors of micro-particles, effectively measuring the velocity and monitoring the motion of micro-particles in the high-speed impact process are two of the primary challenges [21,22,23,24,25,26,27]. Currently, expensive imaging systems with ultra-high fps and resolution are the main solutions for monitoring the high-speed motion of micro-particles [28,29]. However, the strict requirements of the camera and light source make the cost and application threshold excessively high. There is also research into using a laser-delayed light path to obtain a continuous image for a specific time interval. This method demands a laser with a specific wavelength and complex optical delay paths [30]. The advent of modern complementary metal–oxide semiconductor (CMOS) and charge-coupled device (CCD) cameras with fast read-out rates has made it possible to record almost continuous micro-second exposures [31]. Meanwhile, on a nanosecond time scale, the electronically triggered framing cameras enable the collection of multiple frames in a single event (usually at nanosecond exposure times). Although these cameras can capture images at micro-second or even nanosecond intervals, the number of images captured is often small, which makes the analysis of the particles’ motion difficult. Furthermore, particle image velocimetry (PIV) is also used as a typical velocity-measurement method for flow-field analysis [32,33,34]. Although it is applicable to a wide range of scenarios and has high measurement accuracy, it is not suitable for typical industrial fields with tight budgeting requirements for the imaging system [35,36,37,38,39,40,41]. Stroboscopic imaging technology has been applied previously [42,43,44]; however, most of them are restricted to low-speed flow fields that require tracing with a controlled light source, limiting the application and development of this technology. There is little research on the application of this technology in the field of high-speed solid micro-particles’ impact. The commonly used speed-measurement methods mentioned above have stringent requirements for cameras and light sources that are not conducive to widespread popularization and application. Therefore, it is necessary to develop a practical yet economical motion monitoring solution with excellent performance for micro-particles.

Thus, a micro-particle motion monitoring method based on the continuous single-frame multiple-exposure imaging technology (herein called C-smit for short) is proposed in this study. The method in this study adopts high-frequency LED flashes to generate short intervals of illumination together with an excellent low-light performance camera. The effective fps of the captured images for solid micro-particles at high speed can reach up to one million fps. The theoretical minimum observable micro-particle size is less than 10 microns, with a maximum monitoring speed of up to 12 km per second.

## 2. Motion Monitoring Method and Platform Layout

### 2.1. Motion Monitoring Method

**Figure 2:**
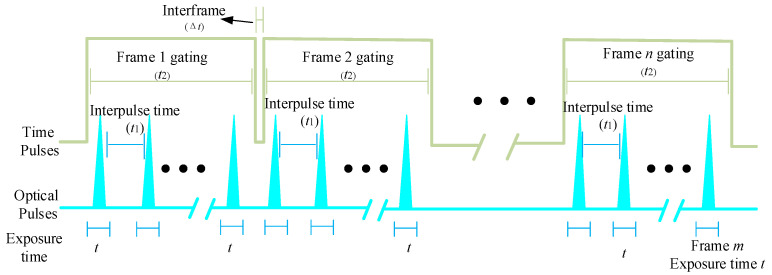
The implementation principle of the C-smit method.

With the aim of obtaining high spatial- and temporal-resolution images using conventional low-cost industrial cameras, the C-smit motion monitoring method adopts high-frequency LED flashes to cut continuous low-fps images. Generally, the motion of high-speed micro-particles taken by a camera with low fps will be blurred for a series of trace points stacked one after another. However, if the flash frequency becomes high enough, the movement distance of micro-particles in each flash period can be observed clearly in one frame image. Therefore, the trajectory and velocity of micro-particles can be captured by the trace points in a single image. The basic implementation principle for the C-smit motion monitoring method is given in Figure 2, that a camera can achieve *n* frames in one second, meaning the fps is *n*. When the camera fps (*n*) was low and the duty cycle ($t_{2}/\left(t_{2}+\Delta t\right)$) was large enough, increasing the frequency (*m*) of the light source led to multiple ($t_{2}/t_{1}$) flash periods within the time ($t_{2}$) of a single frame. As this occurred, high-speed micro-particles passing through the field of view were illuminated multiple times ($t_{2}/t_{1}$) in a single frame. Thus, multiple track points of micro-particles could be recorded in the same frame. If the time (Δt) between frames was small enough, the motion tracks of micro-particles in the field of view could be completely recorded in consecutive images.

In order to ensure the capture rate was greater than 99.5% ($t_{2}/\left(t_{2}+\Delta t\right)\geq 99.5\%$) when micro-particles pass through the field of view of the camera, it was suggested that the product of the actual exposure period ($t_{2}$) and fps (n) of the camera was close to one. For the C-smit motion monitoring method, each pulse intensity of the light source is critical, as this determines the signal-to-noise ratio (SNR) of the imaging system. Moreover, the light source was optimized to accommodate more flash periods ($t+t_{1}$) in each exposure period ($t_{2}$) of the camera, which could reduce the influence of background light for effective micro-particles’ motion capture.

### 2.2. Layout of the Platform

**Figure 3:**
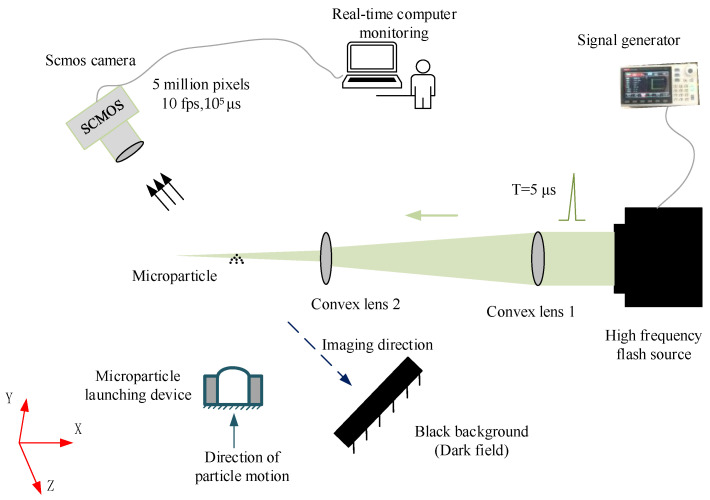
The velocity-measurement platform based on the C-smit method.

The whole layout of the experiment platform consisted of a five-megapixel industrial camera with excellent low-light performance, a high-powered LED, a signal generator, a synchronization trigger device, a computer, a micro-particle launching device, and two convex lenses, as shown in Figure 3. The computer was equipped with programs for altering camera parameter settings and synchronization parameter settings. At the same time, the computer could receive and store images from the camera and perform further image processing. The micro-particle launching device was independently designed and developed in-house based on the principle of a two-stage light-gas gun, which instantly accelerates and ejects micro-particles by way of high-pressure gas. As the pressure increased, the accelerated micro-particles reached speeds of hundreds to thousands of meters per second. The LED light source, including the driving circuit, was designed and manufactured in-house. The lighting cycle could have been as short as 1 microsecond. An industrial camera with a single-pixel readout noise of fewer than 5, quantum efficiency over 80%, and a single-pixel width of 3.75 um was chosen. The signal generator used to control the flash was set with proper pulse width, pulse period, and working current controls. The SNR of images could be improved by adjusting camera gain, light intensity, and exposure time of the light source.

Using the basic layout shown in Figure 3, the operation process is given herein. First, when the platform was operated, the microcontroller triggered the LED to begin high-frequency flashing. The flashes were then focused by two convex lenses across the path of the moving micro-particles. Finally, the focused flashes illuminated the moving micro-particles, allowing the camera to capture their motion. In this way, multiple trace points within a single camera exposure were captured in one image. According to the calibrated motion and set flash period, the velocity of the micro-particles was obtained; the detailed calculation process will be given in Section 3.3.

In this study, the parameters of the equipment adopted are given in Table 1.

## 3. Results and Discussion

### 3.1. Motion Monitoring Experiments of Micro-Particles

A series of micro-particles’ motion monitoring experiments for different micro-particles as given in Table 2 were conducted. The transparent and opaque micro-particles were specially selected to test the platform’s performance in a range of circumstances. Note that due to the inevitable size error in the manufacturing process of micro-particles, the sizes in this study fluctuated within a reasonable range but did not affect the analytical process.

**Figure 4:**
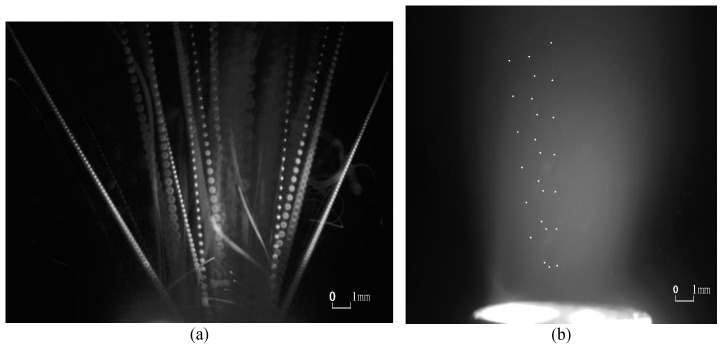
Stainless steel balls with a diameter of 0.5 mm.(**a**) Parameters are set to be camera: 10 fps /cycle:10 us /pulse width 500 ns for many micro-particles. (**b**) Parameters are set to be camera: 10 fps /cycle:10 us /pulse width 500 ns for several micro-particles.

**Figure 5:**
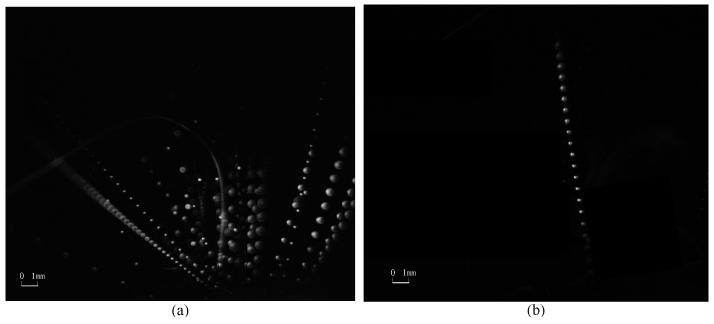
Polystyrene balls with a diameter of 100 μm. (**a**) Parameters are set to be camera: 10 fps /cycle:100 us /pulse width 700 ns for many micro-particles. (**b**) Parameters are set to be camera: 10 fps /cycle:100 us /pulse width 700 ns for several micro-particles.

**Figure 6:**
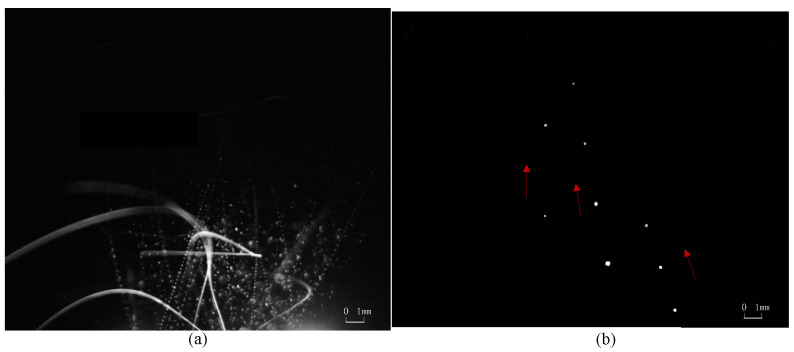
Silica balls with a diameter of 30 to 40 μm. (**a**) Parameters are set to be camera: 10 fps /cycle:100 us /pulse width 700 ns for many micro-particles. (**b**) Parameters are set to be camera: 10 fps /cycle:200 us /pulse width 700 ns for several micro-particles.

**Figure 7:**
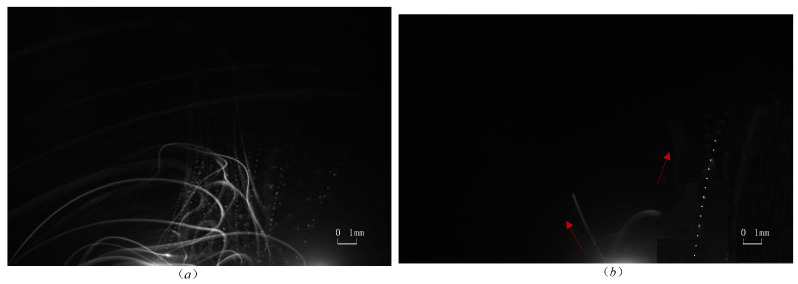
Silica balls with a diameter of 10 μm. (**a**) Parameters are set to be camera: 10 fps /cycle:50 us /pulse width 800 ns for many micro-particles. (**b**) Parameters are set to be camera: 10 fps /cycle:50 us /pulse width 800 ns for several micro-particles.

Typical motion monitoring images using the C-smit method and processed by image-enhancement technology [45,46,47] are given in Figure 4, Figure 5, Figure 6 and Figure 7. It should be noted that the ejected micro-particles may deviate from the set focal length, and thus unfocused micro-particles of the same size may appear to be differently sized. Aiming to validate the monitoring capability of the C-smit method, the number of micro-particles was gradually reduced from a group to just several, and the corresponding parameter settings of camera and light source parameters were adjusted accordingly. It should be noted that only contrast parameters such as gain and aperture of the camera needed to be adjusted, keeping the camera field of view unchanged. In Figure 4, opaque stainless steel balls with a diameter of 0.5 mm were used. From Figure 4a,b, it is found that the high-speed micro-particles’ trace points could be observed with a clear shape in an upward movement. Similarly, for the transparent polystyrene balls of 0.1 mm diameter as shown in Figure 5, the micro-particles trace points were still easy to discriminate, with their outline and movement direction easily observable. Furthermore, smaller transparent micro-particles with diameters of 30–40 μm and 10 μm were tested, and the corresponding motion-trace points in every flash period were still clearly observable, as shown in Figure 6 and Figure 7, respectively.

In summary, the images taken demonstrated that the method can achieve motion monitoring for micro-particles as small as 10 μm in diameter, regardless of the transparency of the micro-particles.

### 3.2. Velocity Measurement

The velocities of micro-particles in the impact process can be obtained by measuring the distances between trace points. For the velocity-measurement process, the width of the camera’s field of view is first determined using calibration targets. Then, the motion time between the trace points is determined by the pulse period of the light source. Finally, the peak values of the luminance curve plotted by the calculated trajectory gray map determine the movement distance between every two micro-particles’ trace points.

**Figure 8:**
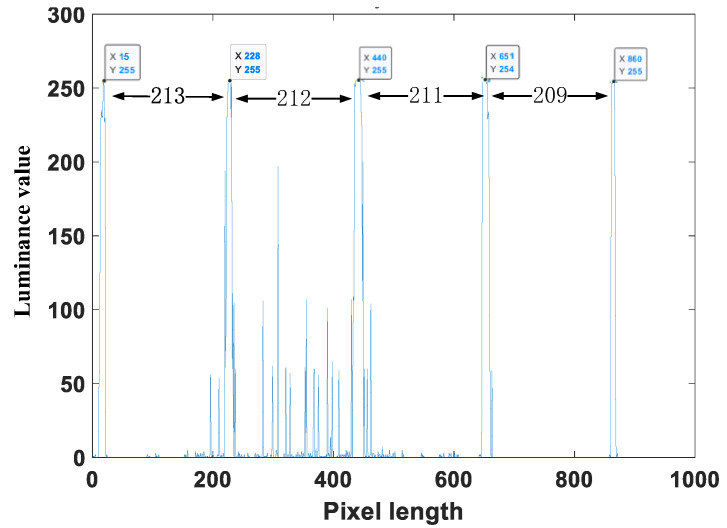
The luminance values of the particle trajectory grayscale.

As an example of the velocity-measurement process of high-speed micro-particles, the last trajectory image in Figure 4b was sampled. First, the actual width of the view field (d) was calculated as 23.437 mm through dimension calibration. Next, the luminance values curve of the particle trajectory as shown in Figure 8 was derived from the grayscale image.

Finally, the luminance values curve in Figure 8 indicates that the dimensionless pixel distances among the luminance peaks are 213, 212, 211, and 209 respectively. Since the dimensionless pixel length $d_{1}$ of the whole image is 2448.002, and the actual field width of view is 23.437 mm, we computed the true distances of each peak as 2.039 mm, 2.029 mm, 2.020 mm, and 2.001 mm respectively based on the scalar relationship. Since the flash period was 10 μs, the ratio of each adjacent peak distance to the flash period was used to calculate the velocity value at each interval. Thus, the velocities in each interval were 209.9 m/s, 203.9 m/s, 202 m/s, and 200.1 m/s, respectively.

Based on the current performance parameters as given in Table 1, the velocity monitoring capability (maximum observable velocity) was roughly deduced by the above calculation process. For the previously analyzed image, there were four periods among five points, and the flight distance was about 2/3 of the total image height. As it only took two trace points to measure the micro-particle velocity and the minimum flash period was set to 1 microsecond, the ratio of the maximum measurable speed ($v_{\max}$) to the current measurement speed was computed as:

(1) $$n=\frac{v_{\max}}{v}=\frac{1}{\frac{2}{3}}\cdot \frac{t}{T_{\min}}\cdot \frac{d}{\frac{d}{4}}=\frac{3}{2}\cdot \frac{10\mu s}{1\mu s}\cdot \frac{4}{1}=60\Rightarrow v_{\max}\approx 12\begin{array}{c} \mathrm{km}/s \end{array}$$

Thus, it was concluded that the theoretical maximum detectable speed was about 12 km/s for 0.5 mm micro-particles with the current equipment.

According to the above calculations, several influencing factors were confirmed to affect the velocity monitoring capability, including the size of the camera view (d) affecting the observable flight distances of micro-particles, the pixel size determining the minimum size of the observable micro-particles, and the flash period (t) affecting the time interval between micro-particle trace points.

It is necessary to point out that the main purpose of this study was to validate the feasibility of the C-smit motion monitoring method, and that the corresponding fps, the monitoring micro-particles sizes, and the motion velocities in this study do not represent the maximum capability of the method. The performance can be effectively improved by improving the resolution of the camera or by reducing the period between flashes.

### 3.3. Discussions

For different micro-particles’ sizes and velocities, the parameters of the camera and flash source needed to be adjusted to obtain clear images. For micro-particles moving at high speed, a light source with high-frequency flashing was necessary to capture more trace points in a limited field of view. On the contrary, for low-speed micro-particles, the flash frequency was limited so that the distances among trace points were large enough to ensure that trace points did not overlap each other. For micro-particles of relatively large size, the trajectory was easily observed even if the flash pulse width was short. However, for smaller micro-particles, flash pulse width needed to be sufficiently long to provide higher luminance, which made the micro-particles’ trajectory more visible.

**Figure 9:**
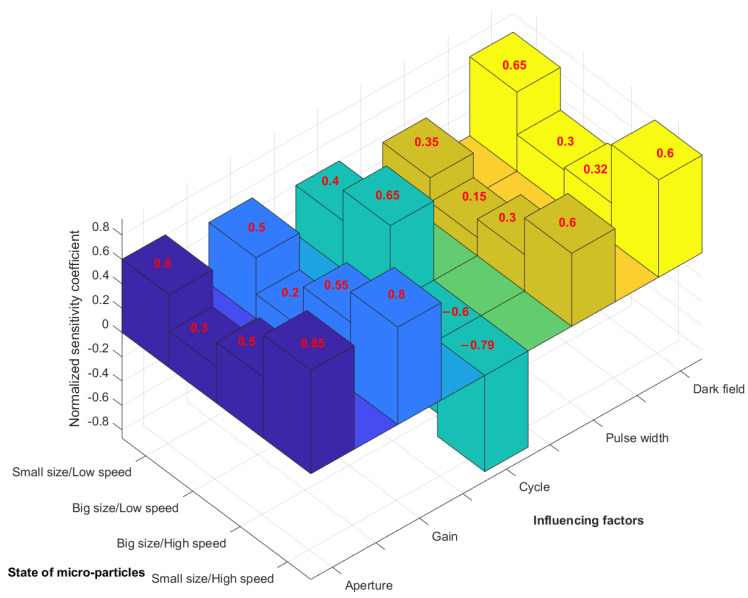
The normalized sensitivity analysis of the imaging results.

Through the adjustment and analysis of every influencing factor by a series of micro-particles motion monitoring experiments, the sensitivity for every factor to the image quality of micro-particles’ trajectory in different cases are evaluated using normalization [48,49]. It should be noted that normalized sensitivity coefficients ranged from −1 to 1, where 1 was the maximum value in the positive correlation and −1 was the maximum value of negative correlation. The micro-particle states were divided into four categories: small-sized micro-particles at low speed, large-sized micro-particles at low speed, large-sized micro-particles at high speed, and small-sized micro-particles at high speed. The influencing factors of camera imaging mainly included: aperture and gain of camera, cycle period and pulse width of light source, and the background dark field. Thus, normalized sensitivity coefficients were obtained through analyzing these five influencing factors under four micro-particles states, as illustrated in Figure 9.

When the micro-particles’ state was small-sized/low-speed, the dark field and aperture had a great influence on the imaging effect, and the sensitivity coefficients of gain, period, light source, and pulse width were small due to the low micro-particle speed. When the micro-particle state was large-sized/low-speed, the situation was similar to the previous state, and the sensitivity coefficients of camera and light source factors were smaller than that of the large micro-particles’ sizes. When the micro-particle state was large-sized/high-speed, the sensitivity coefficient of the light source period was negative (−0.6), because the high-speed state required a smaller light source period to illuminate the moving micro-particles at high frequency. At the same time, improved aperture and gain of camera in this micro-particles’ state were critical. Due to the large micro-particles’ size, images of moving trace points were easily captured, and the sensitivity coefficient of the dark field was small. For the small-sized/high-speed situation, the requirements of all the influencing factors on the motion monitoring imaging were most stringent. The light source period was required to be the smallest to ensure a higher frequency of the flashes, and the corresponding sensitivity coefficient was −0.79. The sensitivity coefficients of aperture, gain, pulse width of light source, and the dark field were 0.85, 0.8, 0.6, and 0.6 respectively. Therefore, according to the normalized sensitivity coefficients in Figure 9, camera parameters, light source parameters, and the dark field conditions were adjusted accordingly for specific micro-particles’ conditions to obtain clear motion trajectories.

Consequently, suggestions for setting the camera and light source of the proposed method are listed in Table 3, based on the sensitivity analysis in Figure 9.

Theoretically, when the micro-particles’ size is smaller than the camera pixel size, its size profile may not be definitively captured by the camera even with the light source and camera parameters adjusted to their optimal states. However, the C-smit method enabled the monitoring of micro-particles smaller than camera-pixel-size due to its insensitivity to focus.

**Figure 10:**
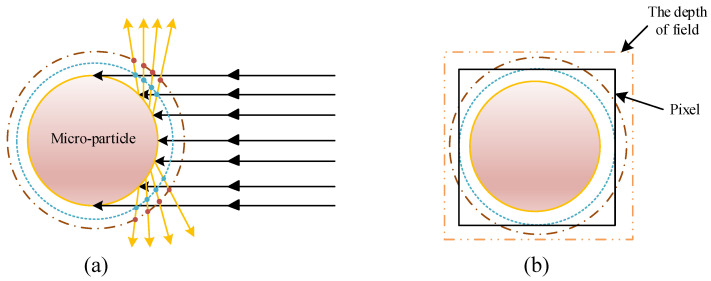
Micro-particle motion monitoring imaging. (**a**) Reflection of light source on micro-particle surface. (**b**) Imaging state after reflection of micro-particles with particle size smaller than pixel size.

As illustrated in Figure 10, incident light reflecting on the micro-particles’ surface (golden circle with solid line) had a corresponding spherical outline (blue circle with dotted line and red circle with dot–dash line) on different focal planes. The brightness value of the actual contour was the largest, and the farther out the image was, the more blurred and less bright it was. As shown in Figure 10b, if the micro-particle size was smaller than the pixel size (black box with solid line), the actual contour of the micro-particle could not be captured and displayed by the pixel. If there was a reflection contour (red circle with dot–dash line) larger than the pixel size within the depth of field (yellow box with double dot–dash line), then the camera pixel captured the reflection contour. Since the circular contours generated by the reflection of micro-particles were concentric spheres, the motion monitoring of micro-particles could be accurately accomplished no matter whether or not the imaging contour was consistent with the actual contour. Therefore, the actual limit of the size for a detectable micro-particle was determined by the pixel size and depth of field of the camera.

## 4. Conclusions

A motion monitoring method based on the continuous single-frame multiple-exposure imaging technology has been proposed. The C-smit method can monitor the motion of high-speed micro-particles using a camera with low fps and a high-frequency LED light source. The study showed that the method can clearly capture the trace points of micro-particles as small as 10 μm in size at high speed, regardless of whether the particles are transparent or not. Based on the current equipment adopted in this study, the effective fps can reach up to one million fps, with a corresponding minimum observable micro-particle size of smaller than 10 μm, up to a maximum theoretical observable monitoring velocity of 12 km/s. Moreover, it was demonstrated that the micro-particles’ motion monitoring capability can be effectively improved by changing the resolution of the camera and the minimum period between flashes without specialized equipment.

Through the normalized sensitivity analysis of the influence factors, it was found that effective motion monitoring for high-speed micro-particles in a specific scene state can be realized by rapid and simple adjustments, according to the sensitivity analysis results. Furthermore, the C-smit method enables the monitoring of micro-particles smaller than the camera-pixel-size due to its insensitivity to focus. Even as the micro-particles’ state changes, high-frequency imaging can be conduct by adjusting only the frequency of the light source. In contrast, other methods require complex adjustment for the magnification and resolution parameters of high-speed camera equipment.

This study provides a convenient and efficient solution with excellent performance for the motion monitoring of micro-particles at high speed, which is realized by using ordinary equipment and basic tuning operations. It is believed that this method will effectively contribute to the development of micro-particles impact research in potential applications, such as transdermal drug delivery in biomedicine, space debris protection, and additive manufacturing.

## Figures and Tables

**Table 1:** Parameter settings of experimental platform.

Equipment	Parameter Settings	Objective
Camera	✧ Capture rate > 99% ✧ FPS: 10 ✧ Gain: 20	Improve SNR
Camera lens	✧ 35 mm f2 macro lens/0.5 magnification	Ensure depth of field
LED	✧ Instantaneous electrical power/current: 1000 watts/150 amperes	Ensure light intensity
Signal generator	✧ Min. pulse signal period: 1 μs ✧ Min. exposure time: 80 ns	High-flash frequency
Convex lens	✧ Focusing module with same focal length	Improve light intensity
Environment	✧ Dark field ✧ No interference in the field of view	Enhanced contrast

**Table 2:** Properties and parameters of micro-particles.

Material	Characteristic	Size (μm)
304 stainless steel	Metal/opaque/spherical	500
Polystyrene	Polymer/transparent/spherical	100
Silica	Inorganic/transparent/spherical	30~40
		10

**Table 3:** The C-smit method settings for different micro-particles’ states.

Micro-Particles States	Camera Settings	Light Source Settings
Small size/Low speed	Large aperture/Large gain	Low frequency/Big pulse width
Large size/Low speed	Large aperture/Small gain	Low frequency/Small pulse width
Large size/High speed	Large aperture/Small gain	High frequency/Small pulse width
Small size/High speed	Large aperture/Large gain	High frequency/Big pulse width

## Data Availability

Data from this study are available from the corresponding author upon a reasonable request.

## References

[1] Goren S.L., John W., Wall S., Wang H.C. (1990). Elastic flattening and particle adhesion. Aerosol Sci. Technol..

[2] Xie J. (2017). Study on Dynamic Characteristics of Micro-Scale Particles Impacting Flat Surface.

[3] Moshtaghi M. (2021). Role of ultrasonic shot peening in environmental hydrogen embrittlement behavior of 7075-t6 alloy. Hydrogen.

[4] Johnson K.L. (2001). Contact Mechanics.

[5] Xue W.J., Liu L.S., Wang K.Y., Wang L., Yang Y.P. (2014). Application and development of shot peening technology. J. Mater. Prot..

[6] Konstandopoulos A.G. (2006). Particle sticking/rebound criteria at oblique impact. J. Aerosol Sci..

[7] Niku-Lari A. (1987). Overview on the shot peening process-sciencedirect. Adv. Surf. Treat..

[8] Hansen J.S., Adler W.F. (1979). Relative erosion resistance of several materials. Erosion: Prevention and Useful Applications.

[9] Heuer V., Walter G., Hutchings I.M. (1999). A study of the erosive wear of fibrous ceramic components by solid particle impact. Wear.

[10] Wang S.B. (1993). Research on particle Erosion problem. Propuls. Technol..

[11] Zhang H.X. (1990). Similarity law of Particle Erosion and Experimental Simulation problem. Chin. J. Aerodyn..

[12] Anderson C.E. (2017). Analytical models for penetration mechanics: A Review. Int. J. Impact Eng..

[13] Mu Z.C., Zhang W., Wang W., Jiang N., Wu X. (2019). Revising the penetration behavior of concrete-like and metal-like materials against the rigid projectile impact. Mech. Mater..

[14] McDonnell J.A.M. (1999). HVI phenomena: Applications to space missions. Int. J. Impact Eng..

[15] Signetti S., Heine A. (2020). Characterization of the transition regime between high-velocity and hypervelocity impact: Thermal effects and energy partitioning in metals. Int. J. Impact Eng..

[16] Kiselev S.P., Kiselev V.P. (2002). Superdeep penetration of particles into a metal target. Int. J. Impact Eng..

[17] Kozorezov K.I., Maksimenko V.N., Usherenko S.M. (1981). Effects of interaction of discrete microparticles with a solid body. Selected Problems of Modern Mechanics.

[18] Burkoth T.J., Bellhouse B.J., Hewson G., Longridge D.J., Muddle A.G., Sarphie D.F. (1999). Transdermal and transmucosal powdered drug delivery. Crit. Rev. Ther. Drug Carr. Syst..

[19] Li W.Y., Cao C.C., Yin S. (2020). Solid-state cold spraying of Ti and its alloys: A literature review. Prog. Mater. Sci..

[20] Li W., Zhang D., Huang C., Guo X. (2016). Application and Research Status of cold spraying technology in additive manufacturing and repair and remanufacturing. Weld. Join..

[21] Assadi H., Kreye H., Gartner F., Klassen T. (2016). Cold spraying-A materials perspective. Acta Mater..

[22] Dewar M.P., Mcdonald A.G., Gerlich A.P. (2012). Interfacial heating during low-pressure cold-gas dynamic spraying of aluminum coatings. J. Mater. Sci..

[23] Kim K.H., Watanabe M., Kuroda S. (2010). Bonding mechanisms of thermally softened metallic powder particles and substrates impacted at high velocity. Surf. Coat. Technol..

[24] Mostafa H.G., David V., Nelson K.A., Schuh C.A. (2018). In-situ observations of single micro-particle impact bonding. Scr. Mater..

[25] Li W.Y., Cao C.C., Wang G.Q., Wang F., Xu Y., Yang X. (2019). ‘Cold spray+’ as a new hybrid additive manufacturing technology: A literature review. Sci. Technol. Weld. Join..

[26] Mostafa H.G., David V., Champagne V.K., Nelson K.A., Schuh C.A. (2018). Response to comment on Adiabatic shear instability is not necessary for adhesion in cold spray. Scr. Mater..

[27] Hassani-Gangaraj M., Veysset D., Nelson K.A., Schuh C.A. (2018). Melt-driven erosion in micro-particle impact. Nat. Commun..

[28] Kirugulige M.S., Tippur H.V., Denney T.S. (2007). Measurement of transient deformations using digital image correlation method and high-speed photography: Application to dynamic fracture. Appl. Opt..

[29] Ball K., Smith G.W., Burt D.J. (2005). Proceedings of SPIE.

[30] Lee J.-H., Loya P.E., Lou J., Thomas E.L. (2014). Dynamic mechanical behavior of multilayer graphene via supersonic projectile penetration. Science.

[31] Veysset D., Lee J.H., Hassani M., Kooi S.E., Nelson K.A. (2021). High-velocity micro-projectile impact testing: A review. Appl. Phys. Rev..

[32] Robert J.D., Bernard F.S. (1975). Integral Engine Inlet Particle Separator: Volume II Design Guide.

[33] Barone D., Hawkins J., Loth E. Efficieney of an Inertial Particle Separator. Proceedings of the 51st AIAA Aerospace Sciences Meeting, No. ALAA.

[34] Wang C., Lin J., Yamamoto F. (2001). Two-dimensional PIV image processing algorithm. J. Hydrodyn..

[35] Hain R., Kahler C.J. (2007). Fundamentals of multi-frame particle image velocimetry (PIV). Exp. Fluids.

[36] Hain R., Kahler C.J., Tropea C. (2007). Comparison of CCD, CMOS and intensified cameras. Exp. Fluids.

[37] Huang H., Dabiri D., Gharib M. (1997). On errors of digital particle image velocimetry. Meas. Sci. Technol..

[38] Kahler C.J., Scholz U., Ortmanns J. (2006). Wall-shear-stress and near-wall turbulence measurements up to single pixel resolution by means of long distance PIV. Exp. Fluids.

[39] Keane R.D., Adrian R.J. (1990). Optimization of particle image velocimeters. Part I: Double pulsed systems. Meas. Sci. Technol..

[40] Keane R.D., Adrian R.J. (1992). Theory of cross-correlation analysis of PIV images. Appl. Sci. Res..

[41] Kirimoto K., Nishio S. Development of higher-order analysisfor multi-frame time-resolved PIV. Proceedings of the 12th International Symposium on Flow Visualization.

[42] Ramer E.R., Shaffer F.D. (1992). Automated analysis of multiple-pulse particle image velocimetry data. Appl. Opt..

[43] Salah N., Godard G., Lebrun D., Paranthoën P., Allano D., Coëtmellec S. (2008). Application of multiple exposure digital in-line holography to particle tracking in a Bénard-von Kármán vortex flow. Meas. Sci. Technol..

[44] Khalighi B., Lee Y.H. (1989). Particle tracking velocimetry: An automatic image processing algorithm. Appl. Opt..

[45] Li N., Liu Y., Han G. (2010). Application of histogram equalization in digital image processing. Gansu Sci. Technol..

[46] Huang C. (2014). Improvement of Histogram Equalization Image Enhancement Algorithm and Implementation in OpenCL.

[47] Gonzalez R.C., Woods R.C. (2017). Digital Image Processing.

[48] Luo H., Wang W., Fu J., Jiao L. (2019). Finite element model updating of satellite sailboard based on sensitivity analysis. Shock. Vib..

[49] Bakir P.G., Reynders E., Roeck G.D. (2007). Sensitivity-based finite element model updating using constrained optimization with a trust region algorithm. J. Sound Vib..

